# Highly pathogenic or low pathogenic avian influenza virus subtype H7N1 infection in chicken lungs: small differences in general acute responses

**DOI:** 10.1186/1297-9716-42-10

**Published:** 2011-01-18

**Authors:** Johanna MJ Rebel, Ben Peeters, Helmi Fijten, Jacob Post, Jan Cornelissen, Lonneke Vervelde

**Affiliations:** 1 Central Veterinary Institute, PO box 65, 8219 PH Lelystad, The Netherlands; 2 Utrecht University, Faculty Veterinary Medicine, Dept. Infectious Diseases and Immunology, Yalelaan 1, 3584 CL Utrecht, The Netherlands

## Abstract

Avian influenza virus can be divided into two groups, highly pathogenic avian influenza virus (HPAI) and low pathogenic avian influenza virus (LPAI) based on their difference in virulence. To investigate if the difference in clinical outcome between LPAI and HPAI in chickens is due to immunological host responses in the lung within the first 24 hours post infection (hpi), chickens were infected with LPAI or HPAI of subtype H7N1. Virus was found in the caudal and cranial part of the lung. With LPAI, virus was localised around the intrapulmonary bronchus and secondary bronchi. In sharp contrast, HPAI was detected throughout the whole lung. However, based on viral RNA levels, no quantitative difference was observed between LPAI and HPAI infected birds. In infected areas of the lungs, an influx of CD8α+ cells as well as KUL01+ macrophages and dendritic cells (DC) occurred as fast as 8 hpi in both infected groups. No major difference between LPAI and HPAI infected birds in the induction of cytokines and interferons at mRNA level in lung tissue was found.

In conclusion, the differences in lethality for chickens infected with LPAI or HPAI could be ascribed to difference in location of the virus. However similar amounts of viral RNA, similar cytokine mRNA levels, and similar influxes of CD8α+ and KUL01+ macrophages and DC were found between HPAI and LPAI in the lungs. A cytokine storm at mRNA level as described for mammals was not observed in the lungs of HPAI infected birds within 24 hpi.

## Introduction

Avian influenza (AI) virus belongs to the Influenza A genus of the orthomyxoviridae family. Over the last years a sharp increase in the number of outbreaks of highly pathogenic avian influenza in poultry and other birds has occurred (OIE). Several of the AI virus subtypes, in particular H5, H7 and H9, have crossed the species barrier and have infected and killed mammals, including humans [1-3]. Therefore, avian influenza is one of the major concerns for public health. Highly pathogenic avian influenza (HPAI) viruses are mainly restricted to H5, and H7 subtypes and infection with these viruses may result in 100% mortality within a susceptible poultry species. Low pathogenic AI (LPAI) viruses can produce respiratory signs such as ocular and nasal discharge and swollen infra orbital sinuses.

One possible hypothesis for the cause of death in mammals due to HPAI infection is the acute induction of high levels of pro-inflammatory cytokines. In humans, acute respiratory viral infection, especially with the H5N1 subtype results in elevated pulmonary concentrations of inflammatory cytokines, a so called cytokine storm, affecting the lungs and subsequently resulted in damage to alveoli and lung tissue resulting in fatal outcome [4,5]. This cytokine storm was also seen in mice [6,7] and macaques [8], and in in vitro infection models [9]. More recently, it was questioned whether lethality is only due to a cytokine storm because mice that lacked CCL2, IL-6 or TNF-α succumbed as often as wild-type mice to infection with a lethal H5N1 virus [10]. Also in humans, an induction of cytokines in the lungs could be found but not in all lethal cases [11]. Little is known about cytokine responses in chickens after HPAI or LPAI infection [12-14]. Especially it is unknown if a cytokine storm develops in the respiratory tract of chickens infected with HPAI and if this is related to the cause of death.

One other hypothesis for the cause of mortality could be that the infection is accompanied by lung immunopathology, which may be mediated by the virus itself or by the host immune response. Whether a difference in influx of macrophages, dendritic cells or lymphocytes and accompanying lung lesions also occur after HPAI or LPAI infection in chickens is not documented.

Also the difference in mortality between HPAI and LPAI infected chickens could be due to the replication site of the virus and the amount of virus generated. In humans it was shown that high amounts of pharyngeal virus resulted in lethality [4].

To investigate whether the clinical differences between HPAI and LPAI infections in poultry is at least partially due to elevated inflammatory responses, it is necessary to use virus strains that are genetically closely related in order to overcome differences in host responses due to viral strain variability. In 1999 in Italy an outbreak of LPAI H7N1 avian influenza occurred among chickens and quails. A mutation of this virus to HPAI H7N1 led to a second outbreak among chickens, turkeys and quails. The resulting epidemic led to the death of 14 million birds and only ended in 2003 after combined efforts of vaccination and stamping out [15,16]. In this study we used two closely related H7N1 strains from the Italian epidemic to investigate the host response specifically in the lung of chickens within 24 h after infection.

The goal of the study was to investigate whether the difference in clinical outcome between HPAI and LPAI infection is due to immune responses in the lung. The localisation of the virus, induction of cytokines and cellular influxes in the lungs were examined in order to investigate the hypothesis that differences in lethality arise due to acute cellular changes and uncontrolled induction of cytokines in the lung.

## Materials and methods

### Virus strains

Avian influenza virus strains A/turkey/Italy/4580/99 H7N1 HPAI and A/chicken/Italy/1067/99 H7N1 LPAI were kindly provided by Dr I. Capua (Istituto Zooprofilattico Sperimentale delle Venezie, Italy). The viruses were passaged once in embryonated eggs. The virus was diluted in sterile phosphate buffered saline to 10^6 ^EID_50_/mL immediately prior to use.

### Animal experiment

Sixty one-day-old male layer type chickens (Lohmann Brown) were obtained from a commercial breeder (Pronk's Broederij, Meppel, The Netherlands). At day 21 of age the chickens were randomly divided in two groups of 30 animals each. At the same time one group of birds received 2 × 10^5 ^EID_50 _of the H7N1 LPAI intranasally and intratracheally, the other group received 2 × 10^5 ^EID_50 _of the H7N1 HPAI. Feed and water were provided *ad libitum*. The study was approved by the institutional Animal Experiment Commission in accordance with the Dutch regulations on animal experimentation.

### Sampling

Six uninfected chickens from each group were used as controls and six infected animals were euthanised at 4, 8, 16 and 24 h post infection (hpi). Of these animals tracheal and cloacal swabs were taken, and gross pathology of the respiratory tract was studied at necropsy. During post-mortem examination, tissues of the respiratory tract were examined for pneumonia and peri-bronchitis associated lessions such as parenchymal hemorrhages and/or other changes in colour or consistency. Cranial and caudal lung, upper and lower trachea were collected for RNA extraction, snap-frozen in liquid nitrogen and stored at -70°C until use. The lung was collected for immunohistochemistry. Samples were fixed immediately by immersion in a zinc salt-based fixative (ZSF), containing 0.5% zinc chloride, 0.5% zinc acetate in 0.1 M Tris base buffer containing 0.05% calcium acetate, pH 7.4 for 48-72 h at room temperature. After fixation, the tissues were dehydrated and embedded in paraffin wax.

### RNA isolation and quantitative RT-PCR

The frozen tissue samples were homogenized (Pro2000 homogeniser, Proscientific, Oxford, USA) in Trizol. RNA was isolated as described previously [17]. Fluid from the swabs (200 μL) was mixed with 300 μL lysis buffer (MagNA Pure LC Total Nucleic Acis Isolaion Kit; Roche Diagnostics Nederland B.V. Almere, The Netherlands) and the recommendations of the manufacturer were followed. A one step RT-PCR reaction was performed to detect the matrix gene of the influenza virus as described previously [18]. For quantification, a standard curve consisting of 10-fold dilutions of A/chicken/Italy/1067/99 H7N1 LPAI virus with a known egg infectious dose in lung tissue homogenate (EID_50_/gram tissue) was used. Data were statistically analysed with treatment and time as variable using ANOVA with the following model

$$\log10{\left(counts\right)}_{ijk}=c+virus_{i}+\beta_{i}vtime_{j}+time+virus.time_{ij}+e_{ijk}.$$

For the quantification of cytokine mRNA, cDNA on RNA of the lungs was made using random hexamer primers and reverse transcriptase. The PCR was employed with on-line detection of the PCR reaction using Syber Green PCR Master Mix (Applied Biosystems, Foster City, CA, USA) in an ABI 7500 Real-Time PCR system (PE Applied Biosystems, Foster City, CA, USA). Quantitative results were determined and normalised with 28S data of the same sample. For the quantification a standard curve of the plasmid with the insert of the cytokine of interest constructed in pGEM-T easy (Promega Benelux b.v. Leiden, The Netherlands) was used. For negative controls, RNA samples without reverse transcriptase in the reaction mixture were used. Primer pairs are given in Table 1.

**Table 1:** Primer sequences as used in the PCR.

RNA target	Primers	Primer sequence (5'-3')	Target accession number
IFN-γ	IFN-γ For IFN-γ Rev	TTCGATGTACTTGGAAATGC TTGCATCTCCTCTGAGACTG	Y07922
28S	28S For 28S Rev	CAAGTCCTTCTGATCGAG TCAACTTTCCCTTACGGTAC	DQ018756
IFN-β	IFN-β For IFN-β Rev	CAGCTCTCACCACCACCTTCTC GGAGGTGGAGCCGTATTCTG	AY974089
IFN-α	IFN-α For IFN-α Rev	TTCAGCTGCCTCCACACCTT TTGTGGATGTGCAGGAACCA	AB255630
IL-6	IL-6 For IL-6 Rev	AGGACGAGATGTGCAAGAAG TGCTGTAGCACAGAGACTCG	EU170468
IL-8	IL-8 For IL-8 Rev	ATTCAAGATGTGAAGCTGAC AGGATCTGCAATTAACATGAGG	DQ393272.2
IL-1β	IL-1β For IL-1β Rev	CAGCACCTCAGCGAAGAG CTGTGGTGTGCTCAGAATCCA	DQ393267.1

Data were analysed for statistical significance by a Mann-Whitney test. Data are expressed as the mean ± standard deviation. A *p *value < 0.05 was taken as the level of significance.

### Immunohistochemistry

Serial 4 μm thick sections were cut from each embedded tissue, collected at 0, 4, 8, 16 and 24 hpi. In short, the samples were deparaffinised, rehydrated, and endogenous peroxidase activity was blocked with hydrogen peroxide 1% in 10% methanol/PBS for 10 min. Sections were incubated with Proteinase K solution (20 μg/mL) in 50 mM Tris, 1 mM EDTA pH 8.0 for 10 min at 37°C in a humidified chamber. Sections were cooled to room temperature for 10 minutes and rinsed three times in PBS for 5 min. Non-specific binding sites were blocked with 0.5% (w/v) bovine serum albumin (PBS/BSA) for 20 min. After washing, the sections were incubated with monoclonal antibodies specific for CD4+ cells, CD8α+ cells or macrophage/monocytes (clones CT-4, CT-8, KUL01 respectively; Southern Biotech, Alabama, USA) in PBS containing 0.1% BSA. A monoclonal antibody specific for viral NP (subtype A; EVS238; EVS) was used to detect AI virus. The markers and the virus were visualised using Dako Real TM EnvisionTM Detection System-HRP (K5007ENV) according to manufacturer's instructions. Slides were counterstained in haematoxylin solution and mounted in Eukitt (Kindler GmbH & Co; Sigma-Aldrich, St. Louis, USA). The haematoxylin-eosin stained tissues were examined for (peri)bronchitis, i.e. presence and extension of bronchiolar epithelial inflammation and peribronchiolar infiltration of inflammatory cells and pneumonia. Due to technical reasons and prioritizing for the different investigation 4 to 6 animals were histologically examined.

## Results

### Clinical signs and gross macroscopical changes

In the first 24 h after infection, no signs of ruffled feathers and depression were observed in the birds infected with LPAI. In the birds infected with HPAI all chickens showed depression at 24 hpi and 4 out of 6 chickens had ruffled feathers. Clinical signs were not seen at earlier time points. During post-mortem examination, tissues were macroscopically scored for alterations, such as hemorrhages on the surface, and diffuse colour changes. In some chickens signs of these abnormalities in lungs and trachea were already found at 8 hpi. No differences during post mortem examination were seen between the HPAI and LPAI infected animals (data not shown).

### Microscopical changes in lungs

Hematoxylin stained lung sections were examined for microscopical changes. The onset of peri-bronchitis and pneumonia was observed as early as 4 h after infection in some of the animals, independent of the virus strain used. The caudal part of the lungs was affected in more animals and earlier after infection as compared to the cranial part. At 16 hpi (caudal part) and 24 hpi (cranial part) all examined animals had signs of pneumonia (Table 2). No major differences in onset and severity of pneumonia or peri-bronchitis between HPAI and LPAI infected animals were observed, although changes in the LPAI infected animals appeared to be more local and localised around the larger airways, whereas changes in the HPAI infected animals appeared to occur throughout the entire lung.

**Table 2:** Number of chickens affected with pneumonia or peri-bronchitis associated lesions at different time points post inoculation (hpi).

	Cranial lung (hpi)				Caudal lung (hpi)			
	
	4	8	16	24	4	8	16	24
Peri-bronchitis								
LPAI	2	0**	1**	3**	5	2*	4*	5*
HPAI	2	1*	2**	1**	4*	3*	5*	2
Pneumonia								
LPAI	1	0**	2**	5*	2	2*	6	4*
HPAI	1**	0*	3	6	3*	3	6	3

N = 6 if not specified otherwise; *n = 5; **n = 4

### Virus detection

**Figure 1:**
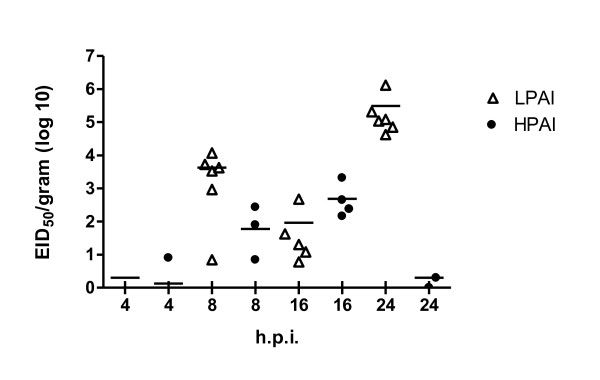
**Scatter plot of the amount of viral RNA of individual birds in the tracheal swabs at different time points post inoculation (hpi) with LPAI (open symbols) or HPAI (closed symbols)**. The horizontal line represents the mean of six individual birds. Viral RNA expression was determined using RT-PCR and data were expressed as EID_50_/g.

At 8 hpi, viral RNA could be detected in tracheal swabs of both infected groups, LPAI 3.6 log_10 _EID_50_/g and HPAI 2 log_10 _EID_50_/g. At 24 hpi in the HPAI infected chickens no animals were positive for viral RNA in the tracheal swabs, while all birds remained positive in the LPAI group (5.5 log_10 _EID_50_/g) (Figure 1). The cloacal swabs were negative for both groups at 4, 8 and 16 hpi. In only one bird of the HPAI group, viral RNA was found in the cloacal swab at 24 hpi (data not shown).

**Figure 2:**
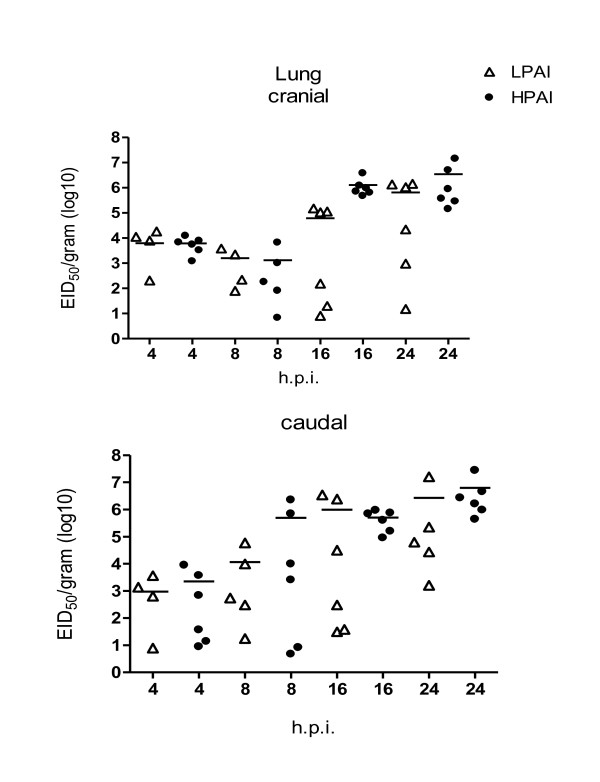
**Scatter plot of the amount of viral RNA of individual birds in the lung at different time points post inoculation (hpi) with LPAI (open symbols) or HPAI (closed symbols)**. The horizontal line represents the mean of 6 individual birds. Viral RNA expression was determined using RT-PCR and data were expressed as EID_50_/g.

Viral RNA was detected in the lungs as early as 4 hpi and the amount of viral RNA increased significantly (F pr < 0.001) upto 24 hpi (Figure 2). In order to investigate the kinetics of the viral RNA in time between HPAI and LPAI infected birds we analysed the data with and without birds that had non-detectable level of viral RNA. When 4 of the 24 LPAI infected birds (2 birds at 4 hpi, 1 at 8 hpi and 1 at 24 hpi) with no detectable level of viral RNA were excluded from the analysis, no significant difference existed between the amount of viral RNA in the caudal lungs of LPAI and HPAI infected birds in time. When the birds with no detectable amount of viral RNA were included in the analysis differences did exist between HPAI and LPAI infected birds. For the cranial part a difference between HPAI and LPAI in time was found (F pr = 0.07), while at 24 h no significant difference existed between HPAI and LPAI. Viral RNA was not detected in the control animals. In the trachea viral RNA was detected from 4 hpi in similar amounts as in the lungs and the amounts increased in time (data not shown).

**Figure 3:**
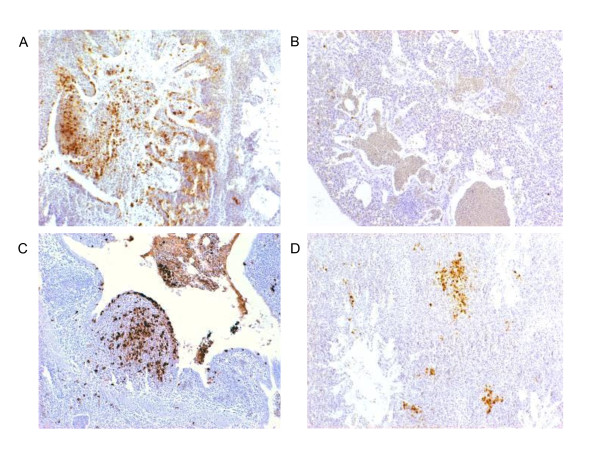
**Detection of AIV (nucleoprotein positive cells) in the lung by immunohistochemistry at 24 hpi (A and C) and 16 hpi (B and D)**. LPAI infected cells are localised around the intrapulmonary bronchus and in adjacent parabronchi (A), but not in the periphery (B). HPAI infected cells are detected around the intrapulmonary bronchus (C) and in peripheral parabronchi (D).

Using immunohistochemistry viral nucleoprotein (NP) was detected in the lung of some of the infected birds at 8 hpi. After infection with LPAI, the NP positive cells were localised around the intrapulmonary bronchus and secondary bronchi. In sharp contrast, NP of HPAI was detected throughout the whole lung around the intrapulmonary bronchus but also in the parabronchi upto the side of the lung where the air enters the airsacs (Figure 3). The number of chickens that had detectable NP in the lungs increased in time (Table 3).

**Table 3:** Number of chickens per group where virus nucleoprotein was detected by IHC at different time points post inoculation (hpi).

Tissue	Cranial (hpi)				Caudal (hpi)			
	
	4	8	16	24	4	8	16	24
Lung								
LPAI	1	0	1	5	0	0*	4	3
HPAI	1	3	6	6	0	1*	6	6
Trachea								
LPAI	0	0	3	4	0	2*	2*	4
HPAI	0	3	5*	6	0	3	5*	5
Airsac								
LPAI	0	2	1**	2				
HPAI	0	3	0**	4				

N = 6 if not specified otherwise; * n = 5; ** n = 4

### Immunological responses

**Figure 4:**
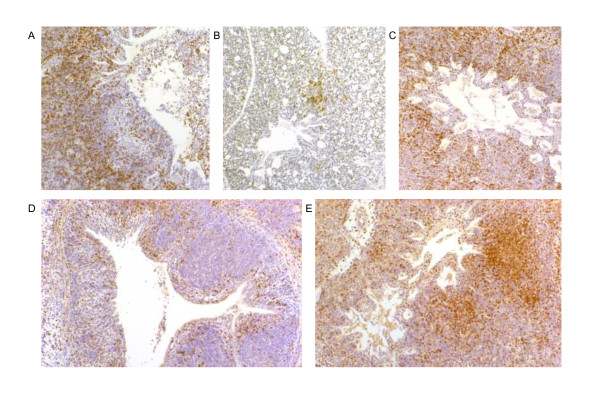
**Detection of CD8α+ (A-C) and KUL01+ (D-E) cells in lung 16-24 h after AIV infection**. In LPAI infected chickens influxes of CD8α+ cells are mainly detected in and adjacent to the intrapulmonary bronchus (A) and rarely in peripheral parabronchi (B). In HPAI infected chickens influxes of CD8α+ are also detected in peripheral parabronchi (C). Influxes of KUL01+ cells were localised in and adjacent to the intrapulmonary bronchus after infection with LPAI (D), whereas KUL01+ cells are detected in peripheral parabronchi after infection with HPAI (E). These cellular influxes co-localised with virus infected cells.

In uninfected birds KUL01+ cells were found scattered throughout the parabronchi and in the lamina propria of the intrapulmonary bronchus and secondary bronchi (Figure 4). Small KUL01+ cell influxes were seen in the interparabronchial septa with both LPAI and HPAI virus as early as 4 hpi, and from 8 hpi the cells changed morphologically from dendritic shape to round with the intensity of expression of KUL01 decreasing. Largest influxes were found in and around the intrapulmonary bronchus in both LPAI and HPAI virus infected birds. Increases in CD8α+ and CD4+ cells were found from 16 hpi onwards (Figure 4). Cellular influxes co-localised with virus infected areas, which resulted in the LPAI virus infected birds in more influxes in the parabronchi adjacent to the intrapulmonary bronchus, whereas in the HPAI virus infected birds influxes were also found in parabronchi closer to the airsacs.

**Figure 5:**
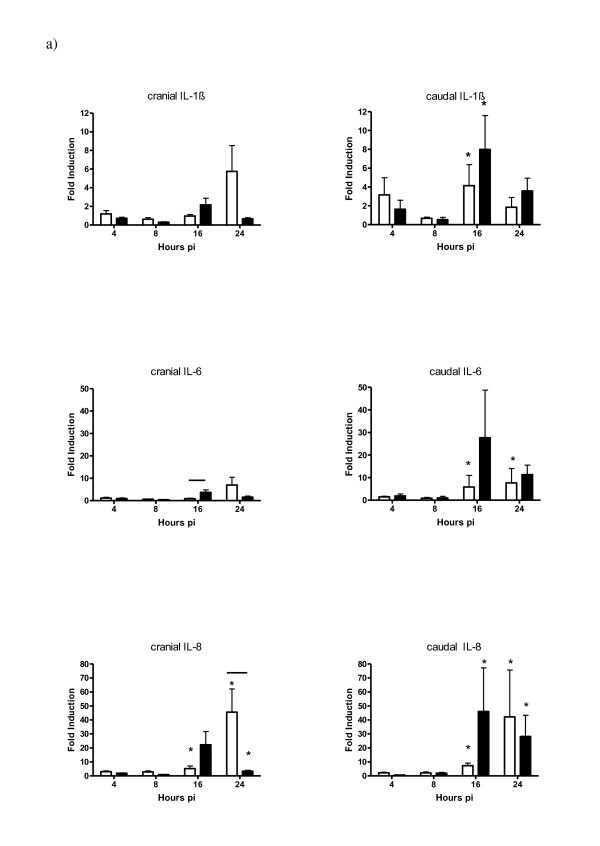
**Pro-inflammatory cytokine mRNA expression in the cranial and caudal part of the lung after inoculation with LPAI (white bar) or HPAI (black bar) at different time points post inoculation (hpi)**. IL-1β, IL-6 and IL-8 mRNA expression was shown as fold induction compared to uninfected birds ± SD. * p < 0.05 between infected and control chickens; line p < 0.05 between the LPAI and HPAI group of a specific time point pi.

**Figure 6:**
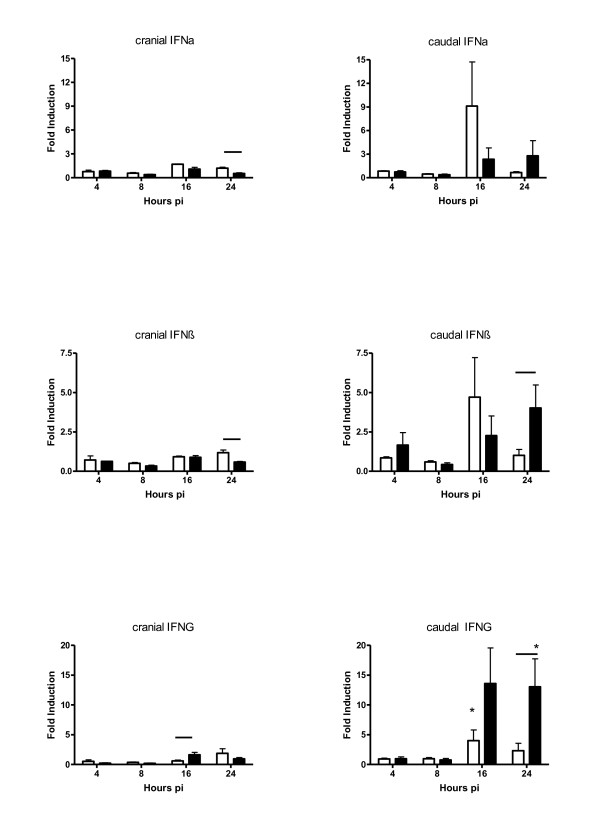
**Interferon mRNA expression in the cranial and caudal part of the lung after inoculation with LPAI (white bar) or HPAI (black bar) at different time points post inoculation (hpi)**. IFN-α, IFN-β and IFN-γ mRNA expression was shown as fold induction compared to uninfected birds ± SD. * p < 0.05 between infected and control chickens; line p < 0.05 between the LPAI and HPAI group of a specific time point pi.

The amounts of pro-inflammatory cytokines and interferons were examined by measuring mRNA levels in the lung (Figure 5 and 6). In the caudal parts of the lung IL-1β, IL-6 and IL-8 mRNA was upregulated compared to uninfected birds, while in the cranial part only induction of IL-8 mRNA was observed. In HPAI infected birds, the mRNA expression seemed to peak at 16 hpi, whereas for the LPAI infected birds highest expression was detected at 24 hpi. The only significant difference (P = 0.06) between LPAI and HPAI infection was a higher IL-8 mRNA induction in the cranial part of the LPAI infected birds (45.5 fold induction in the LPAI group and 3.4 fold induction in the HPAI group).

The IFN-γ mRNA expression (Figure 6) was also upregulated in the caudal part of the lung compared to uninfected birds. At 24 hpi a significant higher induction (2.3 fold induction in the LPAI birds and 13.0 fold induction in the HPAI birds) of IFN-γ was observed in the caudal part of the HPAI infected birds compared to LPAI infected birds.

No significant induction of IFN-α and IFN-β mRNA in the infected birds was observed compared to control birds. In HPAI virus infected birds IFN-α and IFN-β mRNA expression were either equal or lower than in LPAI virus infected birds in the cranial part of the lungs, especially at 24 hpi (for IFN-α 1.2 fold induction in the LPAI birds and 0.5 fold induction in the HPAI birds with p = 0.03; for IFN-β 1.2 fold induction in the LPAI birds and 0.6 fold induction in the HPAI birds with p = 0.06). While in the caudal parts of the lung a different expression pattern was found. At 8 and 16 hpi lower IFN-α and IFN-β mRNA expression was found in HPAI virus infected birds, but at 24 hpi higher expression was detected for IFN-β (1.0 fold induction in LPAI birds and 4.0 fold induction in the HPAI birds with p = 0.06). Thus although no significant difference was found between infected and control birds for some of the cytokine responses a significant difference did exist between both groups. This is due because a small non-significant decreased induction in the LPAI group was found compared to the controls, while a small increase was found in the HPAI group. This led to a significant difference between the groups. The rRNA of 28S which was used for normalisation was constant in time. All measurements of 28S rRNA were within two SD of the mean in all time points and groups used.

## Discussion

In this study we examined virus replication and the host responses to virus infection within 24 h after inoculation of chickens with either a low- or a highly-pathogenic H7N1 avian influenza virus. We hypothesized that differences in clinical outcome between HPAI and LPAI are related to host responses that are induced rapidly upon viral entry. In the experimental infection described in this paper, HPAI infected chickens developed clinical signs from 16 hpi onwards, but no clinical signs were seen in the LPAI infected group. This suggests that indeed within 24 h after infection host responses are of importance for clinical outcome.

The presence of the viral nucleoprotein detected using immunohistochemistry and amount of viral RNA was used to detect and quantify viral replication. Although LPAI and HPAI differ in virulence, no significant difference in the replication abilities of both H7N1 strains either in the cranial and caudal lung or in the trachea were observed within 24 hpi. It could be speculated that LPAI had a delay in viral replication because 2 out of 6 birds showed no detectable levels of viral RNA at 4 hpi, and when analysing in time a small difference was observed between HPAI and LPAI. Also in lungs of LPAI virus infected birds the variation in viral RNA was larger than in HPAI virus infected birds. This might again suggest a delay in replication of LPAI virus. On the other hand it could also mean that LPAI is not able to infect cells as rapidly as HPAI virus can. Moreover, based on NP staining viral replication was also detected more rapidly in HPAI virus infected birds which could indicate a delay in replication or a delay in infection. At 24 hpi the number of animals which are positive for NP staining is lower in the LPAI group compared to the HPAI group, also the PCR showed one bird at 24 hpi without detectable viral RNA. This could indicate that LPAI virus is less able to efficiently infect all chickens in contrast to HPAI virus. Infection was intratracheally, a procedure in which the larger droplets are deposited in the trachea and in the cranial part of the intrapulmonary bronchus, whereas the smallest droplets are deposited deeper into the lung and the airsacs [19,20]. The viral replication started where virus deposition occurs at the bifurcations of the intrapulmonary bronchus up to the secondary bronchi as was also shown by Reemers et al. [12]. Thus, in contrast to mammals, no restriction of viral infection in the chicken lung is observed with these HPAI and LPAI H7N1 strains [21,22].

At 24 hpi viral RNA was not found in tracheal swabs of the HPAI infected birds while in all birds of the LPAI group the tracheal swabs were positive. However at 24 hpi the amount of viral RNA detected in the in tracheal tissue of HPAI and LPAI infected birds was similar. It was described previously that virus shedding from the trachea was found in some studies at 1 dpi while in other studies, with other virus strains shedding started later [23].

Viral replication can be correlated with tissue damage in the lung [24]. For avian influenza it remains unclear whether unrestricted viral dissemination in the lung and cytopathic effects of the virus itself or viral replication and induction of inflammatory responses initiated by chemokines and cytokines released by infected cells are the cause of the tissue damage. In our study we found that although no major differences in viral load were found between HPAI and LPAI virus infected birds, the distribution of the virus within the lungs differed substantially. Interestingly, this difference in spread did not result in differences in number of birds with lung lesions, nor in differences in cellular influx or cytokine induction. An intrinsic more severe cytopathic effect of HPAI virus seems therefore less likely.

The cellular influxes of CD4+, CD8α+, and KUL01+ cells co-localised with virus infected areas. KUL01+ cells are macrophages and dendritic cells [25] and the CD8α+ cells are probably NK cells. Thus LPAI and HPAI induce similar cellular influxes in the lungs. It seems that the number of KUL01+ and CD8α + cells decreased in the HPAI infected birds at 24 hpi. Although down regulation of the marker upon activation could also be the cause of lower number of cells detected [26]. A lower number of CD8α+ and KUL01+ cells could relate to the lower levels of IFN-α and -β at 24 hpi in the HPAI group compared to the LPAI group. Because type I interferons could induce the influx of NK cells and macrophages a reduction in interferon levels, as found in this study, could lead to a decreased influx of these cells. This might lead to a difference in clinical outcome if chickens are not able to clear the virus in the lungs. This hypothesis needs to be further investigated because no cytokine levels or cellular influxes were studied after 24 hpi. Thus, within 24 hpi no difference in activation of these cells occurs after LPAI or HPAI infection, and mortality in the HPAI infected group does not seem to be related to these cellular influxes.

The difference between HPAI and LPAI is mainly found in the caudal part of the lung at the induction level of IFN-γ. However, this difference in induction seemed not to result in a difference in cellular influxes. How this could results in mortality is unknown. Induction of the proinflammatory cytokines IL-1β, IL-6, IL-8 and IFN-α mRNA levels were induced due to influenza H7N1 infection in the lungs of these chickens. Other studies did also find induction of pro-inflammatory cytokines. In lungs infected with H9N2 LPAI virus, IL-6, IL-1β and IFN-β mRNA was found to be induced after infection in different parts of the lungs [12]. This cytokine induction was found at 3 days pi. In a chicken monocyte/macrophage cell line, induction of proinflammatory cytokines was also found after infection with H9N2 although IL-6 mRNA was down regulated [27], while in chicken PBMC infected with H11N9 IL-6 mRNA was induced [28]. It can be concluded that cytokine induction is dependent on the viral strain used.

In our study we did find induction of IL-8 mRNA in the cranial and caudal part of the lungs, although no increased induction was found in the HPAI infected birds when compared to the LPAI infected birds. This is in contradiction with reports in mammals where a cytokine storm was observed after HPAI infection [4,29]. However, other reports also suggest that a cytokine storm alone could not explain lesions in the lungs of mammals [9]. In studies where a comparison was made between HPAI and LPAI strains differences in cytokine induction were found within the same experimental models, which were either in vitro or in vivo studies. Cytokine induction in primary human bronchial epithelial after infection with H5N1 strains and H1N1 differed. Cytokine levels of cells which were infected with H5N1 virus were higher compared to H1N1, while no difference in replication of both viruses was observed [9].

In primary human macrophages infected with H1N1 or H5N1, the H5N1 infection resulted in a hyper-activation of IFN-β and TNF-α [30].

In in vivo studies showed that in mice infected with a high and low pathogenic H5N1 strain, higher levels of IL-6 and TNF-α were found at 6 dpi in the lungs of mice infected with highly pathogenic virus, whereas a higher level of IL-1β was detected at 3 dpi in the mice infected with low pathogenic virus [7]. In a study in chickens, LPAI H5N2 was compared to two H5N1 virus strains differing in lethality. In lungs of both HPAI infected chicken the mRNA levels of IFN-α and IFN-β and pro-inflammatory IL-4, IL-6, IL-8, and IL-15 peaked at 24 hpi when compared to the LPAI H5N1 infected chicken [14]. In most studies that compare HPAI and LPAI virus strains in relation with the host response other viral strains then H7N1 were studied. Thus it is most likely that differences in cytokine induction found in this study compared to other studies are due to the viral strains used in different studies or to the fact that here we studied only the first 24 hpi while other studies used different time points pi. However, we can speculate that the difference in mortality between H7N1 HPAI and LPAI in these commercial birds is not related to hypercytokinemia at mRNA level in the lungs. We cannot exclude systemic cytokine induction in the infected chickens but based on our results this would most probably not be due to differences in response in the lung.

In conclusion, we found that H7N1 LPAI and HPAI strains have a similar effect in the chickens with respect to the immunological parameters tested. Although interferon induction in the caudal part of the lung differs, no differences in lesions and viral load were observed. Thus within the first 24 h after infection none of the investigated parameters could be related to the difference in clinical outcome between HPAI and LPAI infected chickens. A cytokine storm at mRNA level was not observed in the lungs of HPAI infected birds within 24 hpi. Experiments to examine the host response to viral infection by means of mRNA expression profiling are currently being performed and are expected to shed more light on the differences in pathogenesis between HPAI and LPAI strains.

## Competing interests

The authors declare that they have no competing interests.

## Authors' contributions

JMJR initiated and coordinated the work described, analysed data and drafted the manuscript. BP participated with virological input and critically read the manuscript. HF carried out cytokine PCRs. JP isolated RNA and carried out viral PCRs. JC carried out immunohistochemistry (IHC). LV drafted the manuscript and analysed IHC data. All authors read and approved the final manuscript.
